# Comparative proteome profiles of *Polygonatum cyrtonema* Hua rhizomes (Rhizoma Ploygonati) in response to different levels of cadmium stress

**DOI:** 10.1186/s12870-023-04162-6

**Published:** 2023-03-20

**Authors:** Rong Song, Bei Yan, Jin Xie, Li Zhou, Rui Xu, Jia Min Zhou, Xiong Hui Ji, Zi Li Yi

**Affiliations:** 1 College of Bioscience and Biotechnology, Hunan Agricultural University, Changsha, 410128 Hunan China; 2 Institute of Agricultural Environment and Ecology, Hunan Academy of Agricultural Sciences, Changsha, 410125 Hunan China; 3 College of Plant Protection, Hunan Agricultural University, Changsha, 410128 Hunan China

**Keywords:** Cadmium stress, DIA technology, *Huangjing*, Rhizome Cd content, Sugar biosynthesis, ATP generation

## Abstract

**Background:**

The *Polygonatum cyrtonema* Hua rhizomes (also known as Rhizoma Polygonati, RP) are consumed for their health benefits. The main source of the RP is wild *P. cyrtonema* populations in the Hunan province of China. However, the soil Cadmium (Cd) content in Huanan is increasing, thus increasing the risks of Cd accumulation in RP which may end up in the human food chain. To understand the mechanism of Cd accumulation and resistance in *P. cyrtonema*, we subjected *P. cyrtonema* plants to four levels of Cd stress [(D2) 1, (D3) 2, (D4) 4, and (D5) 8 mg/kg)] compared to (D1) 0.5 mg/kg.

**Results:**

The increase in soil Cd content up to 4 mg/kg resulted in a significant increase in tissue (root hair, rhizome, stem, and leaf) Cd content. The increase in Cd concentration variably affected the antioxidant enzyme activities. We could identify 14,171 and 12,115 protein groups and peptides, respectively. There were 193, 227, 260, and 163 differentially expressed proteins (DEPs) in D2, D3, D4, and D5, respectively, compared to D1. The number of downregulated DEPs increased with an increase in Cd content up to 4 mg/kg. These downregulated proteins belonged to sugar biosynthesis, amino acid biosynthesis-related pathways, and secondary metabolism-related pathways. Our results indicate that Cd stress increases ROS generation, against which, different ROS scavenging proteins are upregulated in *P. cyrtonema*. Moreover, Cd stress affected the expression of lipid transport and assembly, glycolysis/gluconeogenesis, sugar biosynthesis, and ATP generation.

**Conclusion:**

These results suggest that an increase in soil Cd content may end up in *Huangjing.* Cadmium stress initiates expression changes in multiple pathways related to energy metabolism, sugar biosynthesis, and secondary metabolite biosynthesis. The proteins involved in these pathways are potential candidates for manipulation and development of Cd stress-tolerant genotypes.

**Supplementary Information:**

The online version contains supplementary material available at 10.1186/s12870-023-04162-6.

## Background

The soil of Hunan province is rich in non-ferrous metals. Due to soil acidification in recent years, the activity of heavy metal ions such as cadmium (Cd) have increased which resulted in excess Cd in soil [1]. The main reasons for high Cd accumulation are the discharge of wastewater from zinc-smelting, waste dumping, and the use of fertilizers with high Cd content [2]. This increased Cd accumulation in soil is a serious threat to both the plant and human health [3]. Continued efforts are needed to ameliorate Cd affected soils in Hunan province. Cadmium triggers the generation of reactive oxygen species (ROS) in plants, affects the uptake and transport of nutrition and water, and damages the photosynthetic machinery. These changes weaken the plant to an extent that it may die [4]. Its absorption in the roots causes reduced length, lower root hair formation, alters its architecture, and initiates lateral root formation [5]. The physiochemical effects of Cd on plants include reduced photosynthetic pigment formation [6], changes in protein levels and protease activity [7], increased free amino acids and proline content [8, 9], altered fatty acid composition [10], and increased activities of the ROS scavenging enzymes [11]. Plants tolerate Cd stress by exclusion of Cd to specific plant parts, biosynthesis of hormones and activation of their signaling, activation of antioxidant systems, and production of phytochelatins and proline [7]. Omics studies in different crop plants have shown that Cd stress induces expression changes in genes related to ROS metabolism and scavenging, cell wall modification, ion transport across membrane, glutathione metabolism, and metal transport [12–14]. For example, these Cd tolerance mechanisms have been discovered in a hyperaccumulator of Cd i.e., *Erigeron annus* [15].

*Polygonatum cyrtonema* Hua is a member of the genus *Polygonatum* (*Liliaceae* or *Asparagaceae* according to the APG III classification system). This genus consists of 71 species (http://www.theplantlist.org/browse/A/Asparagaceae/Polygonatum/; accessed on November 04, 2022), many of which are recorded in the Flora of China. The dried rhizomes of *P. sibiricum* Red., *P. kingianum* Coll. et Hemsl. and *P. cyrtonema* are known as Rhizoma Polygonati (RP) or *Huangjing* in China [16]. Rhizoma Polygonati is used in Traditional Chinese Medicine (TCM) for revitalizing “Qi” and improving “Yin”. Its usage reduces the physical fatigue, premature greying of hair, cough, internal heat, and ill effects of many other diseases related to bones. Rhizoma Polygonati also helps in quenching thirst, moistening the lungs, and nourishing kidneys [17, 18]. The extracts from *P. cyrtonema* and *P. sibiricum* offer health benefits such as antiaging, anticancer, and antitumor effects, immunity enhancement, reduced blood sugar and cholesterol levels, and reduced body weight [19]. Due to these health benefits, it has been enlisted as one of the key plants selected for the 100 billion Chinese medicine industry in Hunan Province. Currently, the main source of *P. cyrtonema* is wild populations which are continuously affected by factors such as land use for infrastructure, artificial excavation, and over-harvesting for its utility in traditional medicine. Apart from wild populations, there is also small-scale cultivation in Hunan province, China. The higher Cd level in Henan province is one of many factors that threaten *P. cyrtonema* natural populations as well as farming. To promote *P. cyrtonema* cultivation and increase farmers’ income, efforts are needed to understand the responses of this plant species in Cd affected soils to develop suitable strategies.

Earlier studies on *P. sibiricum* have shown that the antioxidant system (including superoxide dismutase (SOD), catalase (CAT), and peroxidase (POD)) is the main resistance mechanism against Cd stress [20]. However, *P. sibiricum* is a low Cd accumulator and therefore can be safely planted in Cd-contaminated soils [20, 21]. However, such information is limited for *P. cyrtonema.* A study reported that increase in the soil Cd concentration causes its increased uptake and transport that negatively affects plant biomass. Moreover, the superoxide anion as well as malondialdehyde content increased with an increase in Cd concentration [22]. Since, the tuber (RP) is medicinally important organ of the *P. cyrtonema* [16], therefore, it is essential to understand the threshold Cd levels for safe planting. At the molecular level, the exploration of differential changes in protein expression in the underground parts of *P. cyrtonema* can be helpful to highlight the changes involved in Cd absorption and accumulation. Nevertheless, the scarcity of data that can lay the foundation for research on the regulation of Cd stress tolerance in *P. cyrtonema* calls for omics studies. Such data can be utilized for breeding Cd tolerant *P. cyrtonema* varieties and develop strategies for its sustainable production in Cd affected soils.

Here we report the responses of *P. cyrtonema* tubers to four levels of Cd stress i.e., 1 mg/kg, 2 mg/kg, 4 mg/kg, and 8 mg/kg in comparison to 0.5 mg/kg. First, we determined the Cd content in root hairs, rhizome, stem, and leaf of plants grown in the above-mentioned Cd treated soils. Further, we studied the physiological responses (ROS scavenging enzyme activities) by supplementing *P. cyrtonema* plants with different Cd concentrations. Finally, we used DIA proteome analysis to highlight the differential expression of proteins related to different pathways. The results of this study provide technical foundations for future research.

## Results

### Changes in Cd concentration in different tissues of *P. cyrtonema*

**Fig. 1:**
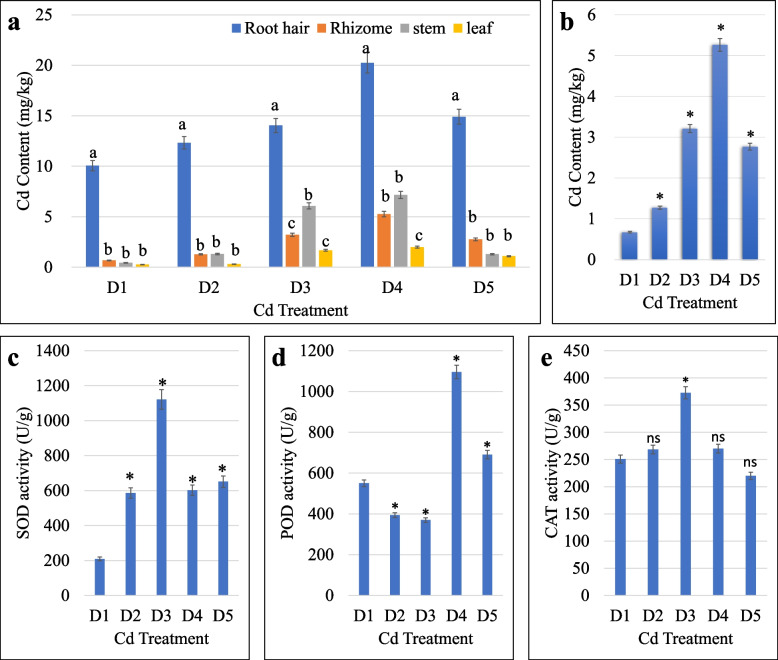
**a** Cadmium concentration in *P. cyrtonema* organs (root hairs, rhizome, stem, and leaf), **b** cadmium concentration in *P. cyrtonema* rhizomes, **c** superoxide dismutase (SOD), **d** peroxidase (POD), and **e** catalase (CAT) activities in *P. cyrtonema* rhizomes harvested from the plants grown in soil with different cadmium contents i.e., D1 (0.5 mg/kg), D2 (1 mg/kg), D3 (2 mg/kg), D4 (4 mg/kg), and D5 (8 mg/kg). The bars represent means (*n* = 3), error bars on the columns represent standard deviation ( ±), and different letters on bars indicate significant differences. * and ns indicate that the differences are statistically significant and non-significant, respectively, as compared to the D1 (*p* < 0.05)

We determined the Cd content of the root hairs, rhizome, stem, and leaf of the four treatment groups i.e., D2-D5 and the control (D1). The Cd content of the root hairs was significantly higher than the other organs (rhizome, stem, and leaf). Overall, the tissue Cd content increased with an increase in soil Cd up to D4 (4 mg/kg), which then slightly decreased in D5 (Fig. 1a). The Cd content did not vary significantly between rhizome, stem, and leaf in D1 and D2 but increased in D3 and D4 significantly. Generally, the Cd content was highest in the root hairs, followed by stem, rhizome, and leaf. Since the tissue of interest for medicinal purposes is mostly the rhizomes, it is important to know the trend of Cd presence under tested conditions. It increased significantly with an increase in soil Cd content up to D4, however, when soil Cd content was further increased to 8 mg/kg (D5), the rhizome Cd content reduced compared to D4 (Fig. 1b).

The increase in soil Cd content (D2-D5) resulted in a significant increase in SOD activity compared to D1. The highest SOD activity was measured in rhizomes grown in D3, whereas it was almost same for D2, D4, and D5. Similarly, the POD activity changed significantly in all treatments; compared to D1, it decreased in D2 and D3 then increased in D4 (maximum). On the other hand, the CAT activity didn’t differ significantly in Cd treatments except D3 compared to D1. Overall, the SOD and POD activities indicate the activation of ROS scavenging enzymes in *P. cyrtonema* rhizomes challenged with Cd stress.

### Proteome analysis of *P. cyrtonema* rhizomes in response to Cd stress

#### Differential proteome expression patterns in response to Cd stress

**Fig. 2:**
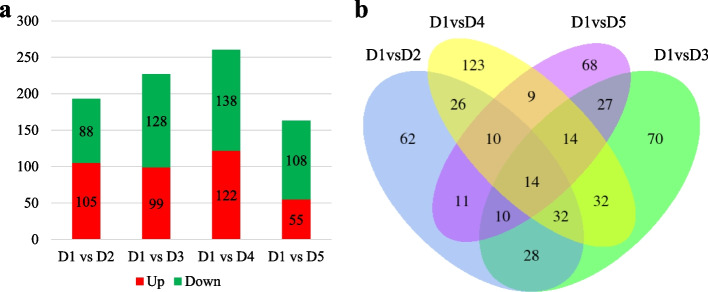
Summary of the differential protein expression in *P. cyrtonema* rhizomes grown in different cadmium concentrations. **a** Number of up/down regulated proteins in cadmium treated (D2-D5) rhizomes as compared to D1. **b** Venn diagram showing number of common and specific proteins that were differentially expressed in rhizomes grown in different cadmium concentrations. Where D1 = 0.5 mg/kg (CK), D2 = 1 mg/kg, D3 = 2 mg/kg, D4 = 4 mg/kg, and D5 = 8 mg/kg

In this study, we compared the proteome of the *P. cyrtonema* rhizomes challenged with different soil Cd concentrations. The true concentration of the samples ranged from 2.304 to 4.58 (average 3.54) µg/µL (Table S1). In total, we identified 14,171 and 12,115 protein groups and peptides through DIA technology, respectively. There were 193, 227, 260, and 163 differentially expressed proteins (DEPs) in D1vsD2, D1vsD3, D1vsD4, and D1vsD5, respectively (Fig. 2a), whereas, 14 proteins were commonly expressed in all treatment comparisons (Fig. 2b). The 173, 87, 239, and 152 DEPs in D1vsD2, D1vsD3, D1vsD4, and D1vsD5 could be annotated in GO. These DEPs were majorly enriched in biological process, followed by cellular component, and molecular function (Fig. S1). On the other hand, 157, 188, 234, and 120 DEPs could be annotated in KEGG database. A higher number of DEPs were enriched in KEGG pathways related to metabolism followed by genetic information processing, cellular processes, and environmental information processing (Fig. S2).

**Fig. 3:**
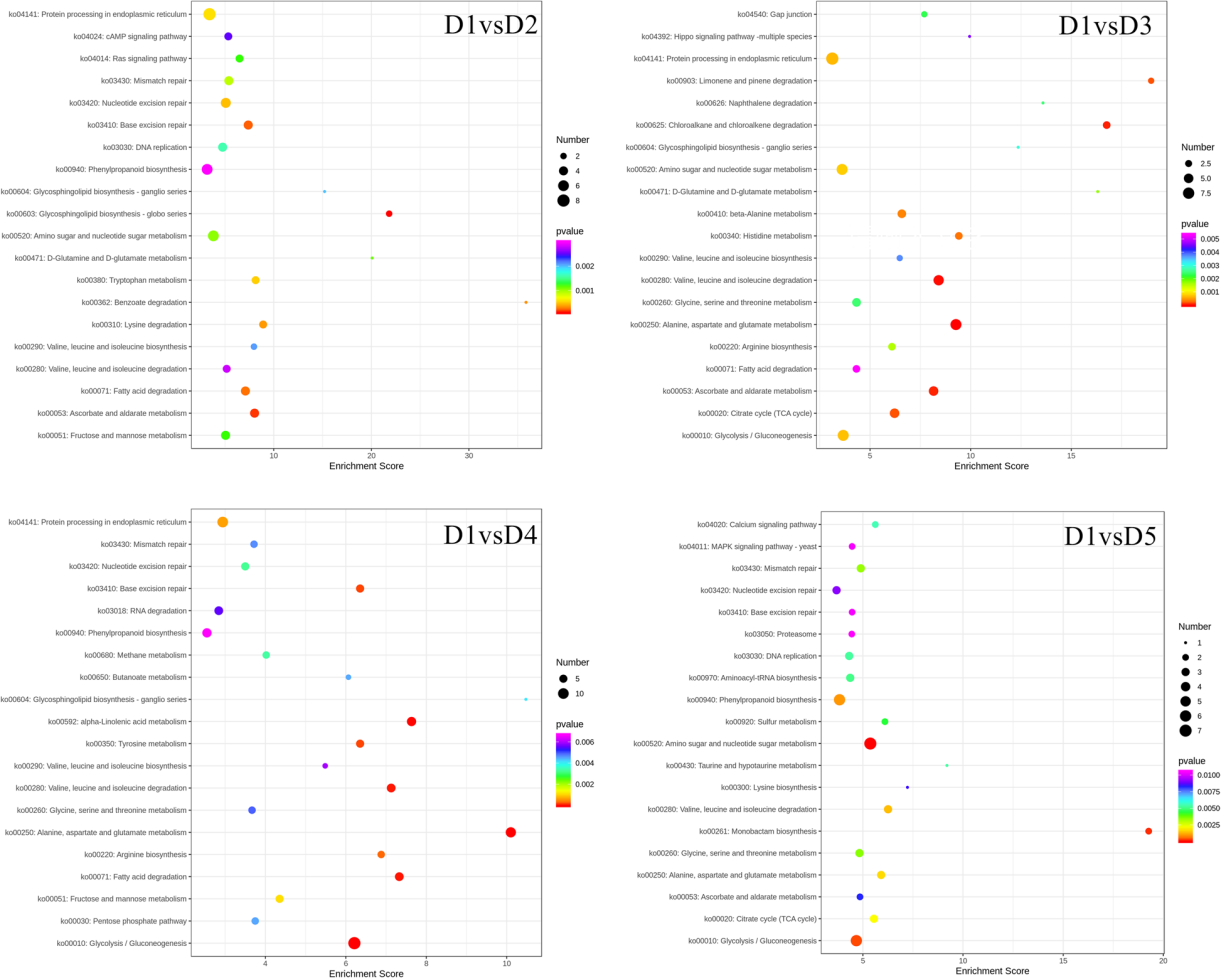
KEGG pathway enrichment analysis. Scatter plots show the KEGG pathways to which the differentially expressed proteins were significantly enriched. Where D1 = 0.5 mg/kg (CK), D2 = 1 mg/kg, D3 = 2 mg/kg, D4 = 4 mg/kg, and D5 = 8 mg/kg

The top five pathways to which the DEPs were enriched in D1vsD2 were glycosphingolipid biosynthesis, ascorbate and aldarate metabolism, base excision repair, fatty acid degradation, and benzoate degradation. Whereas in D1vsD3, the DEPs were significantly enriched in alanine, aspartate and glutamate metabolism, valine, leucine and isoleucine degradation, chloroalkane and chloroalkane degradation, ascorbate and aldarate metabolism, and limonene and pinene degradation pathways. As for D1vsD4, glycolysis/gluconeogenesis, alanine, aspartate and glutamate metabolism, alpha-linolenic acid metabolism, fatty acid degradation, and valine, leucine and isoleucine degradation pathways were top-five significantly enriched. Finally, amino sugar and nucleotide sugar metabolism, monobactam biosynthesis, glycolysis/gluconeogenesis, phenylpropanoid biosynthesis, valine, leucine, and isoleucine degradation were the top-five pathways in which the DEPs were significantly enriched in D1vsD5 (Fig. 3). These enrichment analyses indicate that under the influence of Cd stress, *P. cyrtonema* rhizomes undergo changes related to primary and secondary metabolite biosynthesis, sugar metabolism, and lipid/fatty acid-related changes.

#### Top up/downregulated proteins in *P. cyrtonema* rhizomes grown in different Cd concentrations

The Cd soil content of 1 mg/kg (D2) resulted in an increased expression of glutathione S-transferase (GST, ANH58199.1), RGG repeats nuclear RNA binding protein A-like (XP_020249198.1), uridine kinase-like protein 3 (XP_020247746.1), ketol-acid reductoisomerase, chloroplastic-like (XP_020265027.1), and DNA polymerase delta catalytic subunit (XP_020245108.1) compared to D1. For Cd concentration 2 mg/kg (D3), the proteins that showed highest increase in expression included RGG repeat nuclear binding proteins A- like (XP_020249198.1), inactive leucine-rich repeat receptor-like protein kinase IMK2 (XP_020583190.1), ketol-acid reductoisomerase (KARI, XP_020265027.1), and four uncharacterized proteins. On the contrary, the most downregulated proteins in D2 compared to D1 were peroxidase 51-like (XP_020275593.1 and PKA53650.1), isovaleryl-CoA dehydrogenase 1 (PKA62673.1), ribosomal protein S12, mitochondrial (Q96008.1), and phosphoinositide phosphatase SAC6-like (XP_020247467.1). Whereas, biotin carboxylase 2 (AccC2, PKA64257.1), a rac-like GTP-binding protein ARAC3 (PKA50458.1), plasma membrane ATPase-like (XP_020264658.1), protein BOBBER 2 (PKA58805.1), and beta-hexosaminidase 1 (XP_020272395.1), were downregulated in D3 as compared to D1 (Table 1).

**Table 1:** List of Top-5 up- and downregulated differentially expressed proteins in *P. cyrtonema* rhizomes in response to different levels of cadmium stress

	**Accession**	**Protein Descriptions**	**MW [KDa]**	**log2(FC)**
D1 vs D2	XP_020275593.1	peroxidase 51-like	35.09	-3.69
	PKA62673.1	Isovaleryl-CoA dehydrogenase 1	44.31	-3.39
	PKA66447.1	Thylakoid lumenal 17.4 kDa protein	28.12	-3.21
	PKA53650.1	Peroxidase 51	37.13	-2.99
	Q96008.1	Ribosomal protein S12	14.30	-2.91
	XP_020247467.1	phosphoinositide phosphatase SAC6-like	69.40	-2.91
	XP_020245108.1	DNA polymerase delta catalytic subunit	123.15	3.18
	XP_020265027.1	ketol-acid reductoisomerase-like	57.65	3.61
	XP_020247746.1	uridine kinase-like protein 3	52.65	3.98
	XP_020249198.1	RGG repeats nuclear RNA binding protein A-like		4.34
	ANH58199.1	glutathione S-transferase, partial	13.71	6.13
D1 vs D3	PKA64257.1	Biotin carboxylase 2	130.25	-4.89
	PKA50458.1	Rac-like GTP-binding protein ARAC3	71.57	-4.32
	XP_020264658.1	plasma membrane ATPase-like isoform X1	105.28	-4.22
	PKA58805.1	Protein BOBBER 2	18.08	-3.70
	XP_020272395.1	beta-hexosaminidase 1	60.57	-3.24
	XP_020265027.1	ketol-acid reductoisomerase-like	57.65	3.52
	XP_020583190.1	inactive leucine-rich repeat receptor-like protein kinase IMK2	88.50	3.71
	PKA59025.1	hypothetical protein	40.16	3.73
	ONK73912.1	uncharacterized protein	72.47	3.93
	ONK70065.1	uncharacterized protein		3.94
	ONK57148.1	uncharacterized protein	10.58	4.21
	XP_020249198.1	RGG repeats nuclear RNA binding protein A-like		4.27
D1 vs D4	PKA64257.1	Biotin carboxylase 2	130.25	-5.29
	JE0136	lectin precursor [Galanthus nivalis]	17.09	-4.57
	PKA66447.1	Thylakoid lumenal 17.4 kDa protein	28.12	-3.87
	PKA49849.1	Adenylosuccinate synthetase	50.92	-2.85
	PKA59777.1	Glycine-rich RNA-binding protein	23.09	3.41
	XP_020573354.1	protein-L-isoaspartate O-methyltransferase-like	29.00	3.56
	XP_020265027.1	ketol-acid reductoisomerase-like	57.65	3.74
	XP_020243803.1	glycine-rich RNA-binding, abscisic acid-inducible protein-like	14.64	3.74
	ONK57148.1	uncharacterized protein	10.58	3.94
	ONK62912.1	uncharacterized protein	12.74	6.27
D1 vs D5	PKA66295.1	NADH dehydrogenase [ubiquinone] 1 beta subcomplex subunit 8	13.21	-4.72
	AAS48416.1	cinammate 4-hydroxylase	58.12	-4.44
	PKA56477.1	Bifunctional L-3-cyanoalanine synthase/cysteine synthase C1	39.16	-4.17
	PKA66447.1	Thylakoid lumenal 17.4 kDa protein	28.12	-4.04
	XP_020256866.1	glutamate decarboxylase	56.25	-3.55
	AAT08689.1	lipid transfer protein, partial [Hyacinthus orientalis]	9.17	2.98
	XP_020256259.1	guanine nucleotide-binding protein subunit beta isoform X1	58.38	3.25
	PKA64805.1	14–3-3-like protein GF14 kappa	28.36	3.25
	PKA63043.1	Glutamine–tRNA ligase	81.94	3.73
	PKA50906.1	Ubiquitin carboxyl-terminal hydrolase 12	141.14	6.32

Where, D1 = 0.5 mg/kg, D2 = 1 mg/kg, D3 = 2 mg/kg, D4 = 4 mg/kg, and D5 = 8 mg/kg Cd

Further increase in soil Cd content i.e., 4 mg/kg (D4), induced the upregulation of the AccC2, lectin precursor (JE0136), thylakoid luminal 17.4 kDa protein (TL17, PKA66447.1), and adenylosuccinate synthetase (PKA49849.1) compared to D1. Whereas the highest tested soil Cd content i.e., 8 mg/kg (D5) could induce the upregulation of ubiquitin carboxyl-terminal hydrolase 12 (PKA50906.1), Ggutamine–tRNA ligase (PKA63043.1), 14–3-3-like protein GF14 kappa (GRF, PKA64805.1), guanine nucleotide-binding protein subunit beta isoform X1 (GB1, XP_020256259.1), and lipid transfer protein (AAT08689.1). On the contrary, glycine-rich RNA-binding, abscisic acid-inducible protein-like (XP_020243803.1), KARI (XP_020265027.1), protein-L-isoaspartate O-methyltransferase-like (XP_020573354.1), and several uncharacterized proteins were downregulated in D4 as compared to D1. In case of D5, NADH dehydrogenase [ubiquinone] 1 beta subcomplex subunit 8, mitochondrial (PKA66295.1), cinammate 4-hydroxylase (AAS48416.1), bifunctional L-3-cyanoalanine synthase/cysteine synthase C1 (CYSC1, PKA56477.1), thylakoid lumenal 17.4 kDa protein (PKA66447.1), and glutamate decarboxylase (GAD, XP_020256866.1) were downregulated compared to D1 (Table 1). These results suggest that specific proteins are up/downregulated in *P. cyrtonema* rhizomes grown in different soil Cd concentrations.

#### Protein expression changes in sugar-related pathways and citrate cycle

**Fig. 4:**
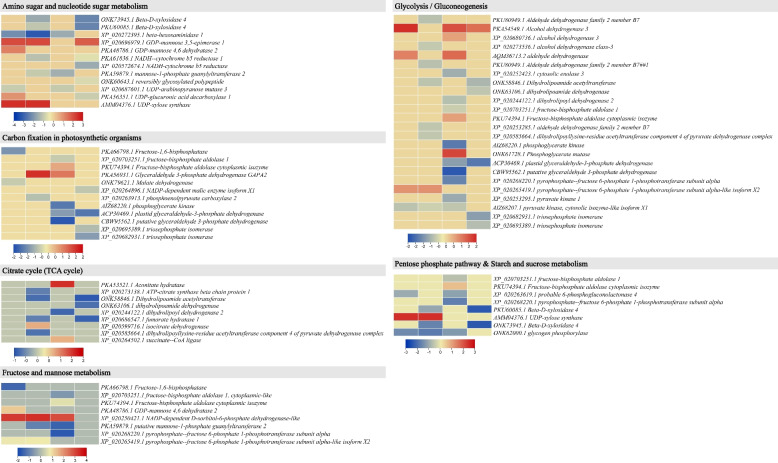
Heatmaps of the log2FC values of the differentially expressed proteins in sugar biosynthesis/metabolism related pathways. The protein IDs are followed by the annotation. The # after the protein names indicate that the protein is common with other pathways and the number after # represents the times it is repeated. The color legends correspond to different pathways

We observed that the number of DEPs enriched in glycolysis/gluconeogenesis pathway increased with an increase in soil Cd content such that 4, 8, 14, and 6 proteins were differentially expressed in D2, D3, D4, and D5, respectively, compared to D1 (CK). Two alcohol dehydrogenases (ALDHs, PKA54549.1 and AQM36713.2) and a phyrophosphate-fructose 6-phosphate 1-phosophtransferase subunit alpha (PEP1, XP_020265419.1) were upregulated in D2, while a pyruvate kinase (PK, AIZ68207.1) was downregulated. Interestingly, an increase in soil Cd content from 1 to 2 mg/kg i.e., D3 resulted in downregulation of seven of the eight DEPs. The downregulated proteins were ALDHs (PKU80949.1, XP_020273536.1, PKU80949.1, and XP_020253285.1), dihydrolipoamide acetyltransferase (DDA, ONK58846.1), dihydrolipoyllysine-residue acetyltransferase component 4 of pyruvate dehydrogenase complex (DLAT, XP_020585664.1), and PK (AIZ68207.1). Whereas, only a PEP1 (XP_020265419.1) was upregulated in D3 compared to D1. A further increase in Cd content (4 mg/kg) i.e., D4 showed contrasting ALDHs expressions as compared to D2 and D3 such that all ALDHs were upregulated. Additionally, a fructose-bisphosphate aldolase (PKA, PKU74394.1) and a phosphoglycerate mutase (PGM, ONK61728.1) were upregulated (Fig. 4; Table S2).

Contrarily, a cytosolic enolase 3 (ENO, XP_020252423.1), dihydrolipoyl dehydrogenase 2 (DHD, XP_020244122.1), fructose-bisphosphate aldolase 1 (ALDOA, XP_020703251.1), phosphoglycerate kinase (PGK, AIZ68220.1), two glyceraldehyde-3-phosphate dehydrogenases (GAPDH, ACP30469.1 and CBW95562.1), a PEP1 (XP_020268220.1), and two PKs (XP_020253295.1 and AIZ68207.1) were downregulated in D4 rhizomes as compared to D1. Finally, D5 caused the downregulation of a DDA (ONK58846.1), dihydrolipoamide dehydrogenase (DLPD, ONK63106.1), GAPDH (ACP30469.1), and triosephosphate isomerase (TPI, XP_020682931.1 and XP_020695389.1) and upregulation of an ALDH (PKA54549.1). These observations indicate that glycolysis pathway is significantly affected by soil Cd content such that an increase in concentrations from 0.5 to 8 mg/kg causes downregulation of major steps in the pathway. The lower number of DEPs in D1vsD5 may indicate that higher Cd content i.e., 8 mg/kg impairs the glycolysis process to an extent that only a limited number of proteins are expressed (Fig. 4; Table S2). The upregulation of ALDHs in most of the treatments indicate interconversions of ethanol to acetaldehyde, which is used in citrate cycle as well as propionate metabolism. Whereas the downregulation of PK indicates that phosphoenolpyruvate conversion to pyruvate is impaired due to Cd stress.

Interestingly, three of the four DEPs enriched in fructose and mannose metabolism i.e., GDP-mannose 4,6 dehydrogenase 2, NADP-dependent D-sorbitol-6-phosphate (S6PDH), and PEP1 were upregulated in D2 compared to D1. The S6PDH was common to D2, D3, and D4, where its expression increased compared to D1. Interestingly, none of these were differentially expressed in D5 compared to D1. In case of pentose phosphate pathway, one and four DEPs were enriched in treatment comparisons D1vsD2 and D1vsD4, respectively, where these DEPs were either downregulated or showed varied expression patterns within a treatment. In case of D-glutamine and D-glutamate metabolism, only glutamate dehydrogenase (GDH, PKA59753.1) was upregulated in D2 and D3 compared to D1. However, it was not differentially expressed in higher Cd concentrations (D4 and D5). As for starch and sucrose metabolism pathway, two, four, one, and two DEPs were enriched in D2, D3, D4, and D5, respectively, compared to D1. Among these, UDP-xylose synthase (AMM04376.1) was upregulated in D2 and D3 but did not show differential expression in D4 and D5 compared to D1. Two beta-D-xylosidase 4 (PKU60085.1 and ONK73945.1) and a glycogen phosphrylase (ONK62000.1) were downregulated in D3 compared to D1 (Fig. 4; Table S2).

Nine DEPs were enriched in citrate cycle. None of these proteins were differentially expressed in D1vsD2. Whereas, four of the five DEPs were downregulated in D3 compared to D1. The downregulated proteins included ATP-citrate synthase beta chain (XP_020273138.1), DDA (ONK58846.1), fumarate hydratase 1 (FH1, XP_020686547.1), isocitrate dehydrogenase (IDH, XP_020599716.1), and DLAT (XP_020585664.1). Whereas, another IDH (XP_020599716.1) was upregulated in D3 compared to D1. In case of D4, an aconitate hydratase (PKA53521.1) and succinate-CoA ligase (XP_020244122.1) were upregulated while a DLD (XP_020264502.1) was downregulated compared to D1. Finally, the FH1, DDA, and DLD were downregulated in D5 compared to D1. These changes are consistent with those of glycolysis related proteins (Fig. 4; Table S2).

Since these DEPs were also enriched in other pathways such as citrate cycle, fructose and mannose metabolism, starch and sucrose metabolism, pentose phosphate pathway, ascorbate and aldarate metabolism, and amino sugar and nucleotide sugar metabolism, therefore, it can be understood that Cd stress (1–4 mg/kg) significantly affects (impairs) the major pathways involved in plant growth and development. Whereas, D5 (8 mg/kg) is too high Cd concentration, which significantly impairs growth and development related process to an extent that only a limited number of DEPs were found.

#### Protein expression changes in alanine, aspartate and glutamate metabolism and related pathways

**Fig. 5:**
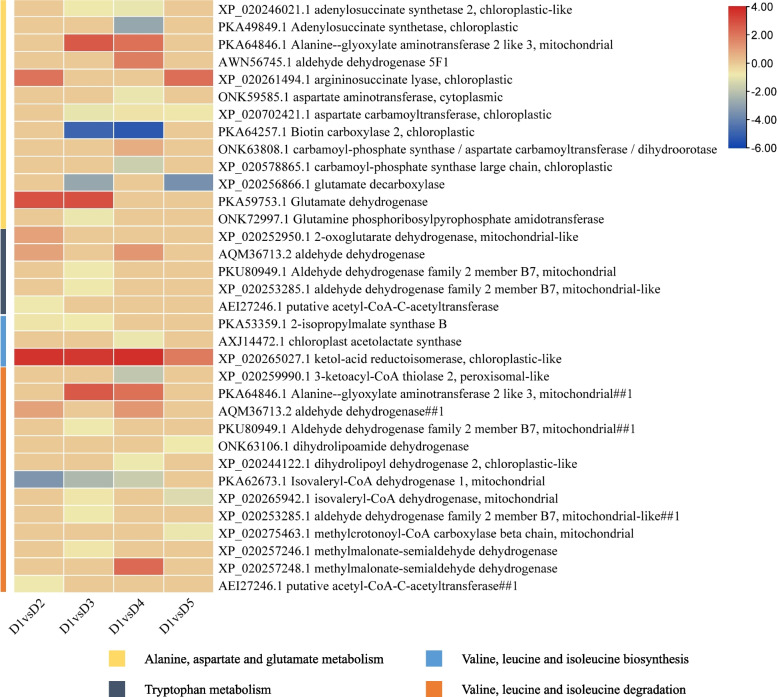
Heatmaps of the log2FC values of the differentially expressed proteins in alanine, aspartate and glutamate metabolism, tryptophan metabolism, valine, leucine and isoleucine biosynthesis/degradation pathways. The protein IDs are followed by the annotation. The # after the protein names indicate that the protein is common with other pathways and the number after # represents the times it is repeated. The color legends correspond to different pathways

Thirteen proteins were enriched in alanine, aspartate and glutamate metabolism. Two DEPs i.e., argininosuccinate lyase (ASL, ONK59585.1) and GDH (PKA59753.1) were upregulated in D2 compared to D1. Whereas, two of the seven DEPs (an alanine-glyoxylate aminotransferase 2 like 3 and GDH) were upregulated in D3 compared to D1. The downregulated DEPs included adenylosuccinate synthetase 2 (ADSS2, XP_020246021.1), aspartate carbamoyltransferase (ATCase, XP_020702421.1), AccC2 (PKA64257.1), GAD (XP_020256866.1), and glutamine phosphoribosylpyrophosphate amidotransferase (GPAT, ONK72997.1). Similar to other pathways, a relatively higher number of proteins (nine) were differentially expressed in D1vsD4. Whereas, only three proteins i.e., ASL (up), ATCase (down), and GAD (down) were differentially expressed in D1vsD5. These expression changes indicate that proteins related to L-aspartate, L-alanine, and oxaloacetate were upregulated in at least one Cd treatment (D3 or D4), while those involved in succinate, glutamate, carbamoyl phosphate, 5-phospho-ribosylamine were downregulated in response to Cd stress (mostly in D3 and D4). The downregulation of glutamate, which was also enriched in D-glutamine and D-glutamate metabolism, was evident from the increased expression of GDH in D2 and D3 (Fig. 5; Table S2).

A protein involved in tryptophan metabolism i.e., 2-oxoglutarate dehydrogenase (2OGDH, XP_020252950.1) was upregulated in D2 compared to D1 but didn’t differentially express in other treatments. On the other hand, an ALDH (AQM36713.2) was upregulated both in D2 and D4 compared to D1, whereas two other ALDHs were downregulated in D3 compared to D1. A putative acetyl-CoA-acetyltransferase (AEI27246.1) was downregulated in D2 compared to D1 (Fig. 5; Table S2).

The DEPs enriched in valine, leucine and isoleucine biosynthesis pathway were mostly downregulated in all treatments with some exceptions such as one KARI (XP_020265027.1) was upregulated in all treatments compared to D1. Contrarily, the ALDHs showed variable expressions patterns (Fig. 5; Table S2). These observations suggest that Cd stress induces expression reduction of proteins involved in L-isoleucine and valine biosynthesis. This proposition is further supported by the fact that proteins enriched in valine, leucine, and isoleucine degradation were also downregulated in response to Cd stress.

#### Protein expression changes in secondary metabolite biosynthesis pathways

The DEPs were enriched in flavonoid biosynthesis (01), isoquinoline alkaloid biosynthesis (01), phenylpropanoid biosynthesis (11), sphingolipid signaling (03), terpenoid backbone biosynthesis (02), tropane, piperdine and pyridine alkaloid biosynthesis (02) pathways. Only one protein annotated as cinnamate 4-hydroxylase (AAS48416.1) was enriched in flavonoid biosynthesis pathway, where it was exclusively repressed in D3 and D5. Whereas one DEP enriched in isoquinoline alkaloid biosynthesis i.e., bifunctional aspartate aminotransferase and glutamate/aspartate-prephenate aminotransferase isoform X1 (PAT, XP_020263572.1) showed reduced expression in D2 and D4. Two DEPs annotated as ALDH (XP_020241182.1) and POD5-like (XP_020586448.1) showed increased expressions in D2 and D4 but didn’t differentially express in other treatments. Interestingly, the three DEPs enriched in sphingolipid signaling pathway i.e., serine/threonine-protein phosphatase 2A 65 kDa regulatory subunit A beta (PPP2RA1, XP_020264363.1), phospholipase D alpha 1 (PLDα1, XP_020690474.2), and a PLDα1-like (XP_020270081.1) were upregulated in D2 and D4 compared to D1. Finally, the terpenoid backbone biosynthesis, phenylpropanoid biosynthesis, and tropane, piperidine and pyridine alkaloid biosynthesis pathway related proteins were downregulated in Cd treated *P. cyrtonema* rhizomes. These protein expression trends indicate that Cd stress causes reduction in secondary metabolite biosynthesis (except sphingolipid pathway proteins) in the studied genotype (Table S2).

#### Protein expression changes in other important pathways

We observed that the proteins enriched in alpha-linoleic acid metabolism showed different expression profiles e.g., ALDs were both up and downregulated. Two proteins i.e., 12-oxophytodienoate reductase 7 and 3-keotacyl-CoA thiolase 2 were downregulated while hydroperoxide dehydrogenase was upregulated in different treatments compared to D1. None of these proteins were differentially expressed in D1vsD5. These changes indicate that Cd stress induces expression changes in proteins related to alpha-linoleic acid metabolism pathway, which possibly changes the accumulation patterns of alpha-linoleic acid (Table S2).

One of the proteins enriched in fatty acid degradation i.e., 3-ketoacyl-CoA thiolase 2 was downregulated in D2-D5, while ALD transcripts showed variable expression trends in response to Cd stress. Interestingly, peroxisomal acyl-coenzyme A oxidase 1 was downregulated in D2 while upregulated in D4 compared to D1. Two other proteins i.e., peroxisomal fatty acid beta-oxidation multifunctional protein isoform X1 and putative acetyl-CoA-C-acetyltransferase were upregulated in D4 and D5 (Table S2). These expression changes indicate that Cd stress affects fatty acid degradation.

The glycosphingolipid biosynthesis – globo series related protein alpha-D-galactosidase was downregulated in D2 and beta-hexosaminidase 1 was downregulated in *P. cyrtonema* rhizomes grown in D2-D4 compared to the ones cultivated in D1 (Table S2). Whereas, only one DEP was enriched in benzoate degradation, which showed reduced expressions in D2 as compared to D1 (Table S2). Likewise, the proteins related to calcium signaling i.e., mitochondrial outer membrane protein porin 1-like and 5-like were downregulated exclusively in D2-D5 compared to D1. Interestingly, all the proteins enriched in base excision repair pathway (DNA ligase 1, DNA polymerase delta catalytic subunit, DNA lyase, and proliferating cell nuclear antigen) were upregulated in response to Cd stress (1 to 8 mg/kg) compared to CK (Table S2). Finally, two serine/thronine-protein kinase SRK7 (up in D2 and D4) and SRK2E (up in D2) were differentially expressed in rhizomes of *P. cyrtonema* plants grown in different levels of Cd stress compared to D1 (Table S2).

Taken together, the comparative protein expression results show that when the Cd stress concentration was 0.5–4.0 mg/kg, the number of DEPs gradually increased with an increase of Cd concentration. Whereas, the Cd of 8.0 mg/kg significantly affected the *P. cyrtonema* rhizomes such that the number of DEPs was significantly lower compared to the lower Cd concentrations (0.5–4.0 mg/kg). These observations suggest that Cd concentration of 4 mg/kg affects the normal functioning of several primary and secondary metabolism related pathways in *P. cyrtonema* rhizomes and a concentration of 8 mg/kg can be devastating (Table S2).

## Discussion

Rhizomes of the *P. cyrtonema* i.e., *Huangjing* are used in TCM [16]. Hunan province is the main *Huangjing* producing area. However, the soil-Cd content of Hunan province farmland is increasing [2]. Moreover, Cd may also accumulate in *P. cyrtonema* edible parts i.e., rhizome, which can be a hazard to human health. Therefore, we measured the accumulated Cd content in *P. cyrtonema* root hair, rhizome, stem, and leaf grown in soils with Cd content ranging from 1–8 mg/kg. The results that rhizomes had higher Cd content (1.2 to 5.2 mg/kg) (Fig. 1a & b) when grown in soils having 1–4 mg/kg Cd are alarming since the Chinese national standard for acceptable Cd concentration in roots and tuber vegetables is 0.2 mg/kg, and in all types of foods is 0.003 to 1.0 mg/kg [23]. Our data indicate that higher soil Cd content will result in increased Cd accumulation in the edible part i.e., rhizome, which can pose serious health concerns in humans. To this regard, the response mechanisms of *P. cyrtonema* rhizomes grown under different soil-Cd concentrations were explored. For this, we used DIA proteome analysis technology and attempted to understand the key proteins and related pathways that are expressed *P. cyrtonema* rhizomes grown in different Cd concentrations.

The increasing number of DEPs with an increase in soil-Cd content up to D4 (4 mg/kg) indicate that DEP number and Cd concentrations are positively correlated to a certain limit. These data suggest that different levels of Cd in soil can induced different numbers of proteins in *P. cyrtonema* rhizome. Similar findings were reported in *Tamarix hispida* [24]. Notable observation was that the D5 affected the *P. cyrtonema* rhizome proteome the most. These results indicate that most pathways were affected to an extent that their function might have been impaired, which can be an indication of reduction in biomass and yield as observed in *Avena sativa, Zea mays, Lupinus luteus, Raphanus sativus,* and *Phacelia tanacaetifolia* [25].

The upregulation of GST in soils with higher Cd content, indicates its possible involvement in conjugation of reduced glutathione to exogenous electrophile. This proposition is based on the fact that this protein plays role against exogenous/endogenous hydrophobic electrophiles and also have detoxification role against certain herbicides [26]. Whereas, the GDH’s upregulation indicates that, similar to other abiotic stresses, under the influence of Cd stress, its activity is increased [27]. We say this because abiotic stresses induce ROS generation, which in this case is evident from the higher activities of SOD and CAT in D2 and D3. Whereas, the downregulation of PODs (POD51 and POD51-like) is consistent with POD activity in respective treatment (Fig. 1). This increased ROS scavenging enzyme activity may signal the expression of GDH to form glutamate under heavy metal (e.g., Ni and Al) stress [28]. The downregulation of protein-L-isoaspartate O-methyltransferase-like protein in D4 together with the lower activities of CAT and SOD suggest that 4 mg/kg Cd damages the antioxidant enzymes, which affects their activities. This proposition is based on the known role of this protein in Arabidopsis i.e., it repairs the isoaspartyl damage to the antioxidant enzymes [29]. Whereas, the upregulation of KARI is an indication of a possible defense response since this gene has been reported to be upregulated in different abiotic stresses [30]. Similarly, the upregulation of AccC2, TL17, and adenylosuccinate synthetase show increased purine biosynthesis, lipid transport or assembly, and acetylation [30, 31]. The upregulation of GRF and GB1 in the highest tested Cd concentration (8 mg/kg) indicates changes in nutrient metabolism, transmembrane signaling, as well as modulation of cell wall under stress [32, 33]. Contrarily, the downregulation of isovaleryl-CoA dehydrogenase 1, ribosomal protein S12, and phosphoinositide phosphatase SAC6-like in D2 and AccC2, ARAC3, ATPase-like, BOBBER 2, and beta-hexosaminidase 1 in D3 can be an indication of impaired/reduced leucine degradation [34], inositol signaling [35], polar growth [36], energy transport across plasma membrane [37], and auxin transport [38]. Since different proteins were downregulated in different Cd stress treatments, therefore, the regulation of these proteins can be used as biomarkers. Further confirmation experiments should be carried out to use these proteins into useful markers. Moreover, the downregulation of ABA-inducible protein and the glycine-rich RNA-binding protein indicate that Cd stress affects the amino acid biosynthesis, the native defense responses of *P. cyrtonema* against abiotic stresses [39], and metal stress responses [40]. Similarly, the expression changes in TL17, CYSC1, and GAD in D5 compared to D1 indicate that lipid transport and assembly [31], cyanide detoxification and its maintenance in *P. cyrtonema* rhizomes [41], and secondary growth of the rhizome is affected by imposed Cd stress [42]. These statements are based on the known functions of the afore mentioned proteins/genes in different plant species [31, 41, 42]. Taken together, these are novel candidate proteins, which are involved in several metabolism and defense related pathways that can be manipulated for different level of Cd stress responses/tolerance.

The downregulation of multiple proteins in carbon fixation in photosynthetic organisms pathway (Table S2) indicates that several downstream pathways such as glycolysis/gluconeogenesis and other sugar related pathways are increasingly affected in *P. cyrtonema* rhizomes by the imposed Cd stress [43]. These observations are important from the perspective that sugar metabolism contributes to the overall plant growth and development and enables plants to survive under abiotic stresses scenarios. Earlier studies in maize and *sassafras* have shown that Cd stress caused decrease in sucrose synthesis-related enzyme activity and its increased hydrolysis [44, 45]. Consistent with this report, the expression changes in the DEPs detected in D2-D5 indicate that Cd significantly impaired the sugar biosynthesis in *P. cyrtonema* rhizome. Similarly, the higher number of downregulated proteins involved in amino sugar and nucleotide sugar metabolism, fructose and mannose metabolism, and pentose phosphate pathways indicates that Cd stress induces reduction in the expression of sugar metabolism related proteins. Thus, our results imply that the sugar contents of *P. cyrtonema* rhizomes grown in higher Cd concentrations would be negatively affected. These propositions are consistent with the earlier work on *sassafras,* which reported that Cd stress affected the inner cell membranes and had photosynthesis repressing affects [45]. Therefore, it is possible that increasing Cd stress would have affected the photosynthesis in leaves, which ultimately resulted in downregulation of the sugar biosynthesis related proteins. Further experimentation in this regard would reveal the key genes contributing towards the expression changes in sugar biosynthesis related proteins. Apart from the main sugar biosynthesis/metabolism pathways, the reduced expression of TCA cycle proteins i.e., aconite hydratase (ACOH), ATP-citrate synthase, DDA, DLPD, DLD, DLAT, and FH1 (Table S2) is an indication of the repression of ATP generation and provision of carbon skeletons for different processes in response to Cd stress [46]. These proteins control essential steps to convert different intermediates to acetyle-CoA, citrate, cis-Aconitate, and isocitrate [47]. In alanine, aspartate, and glutamate metabolism pathway, L-aspartate is converted to oxaloacetate, which is then used in TCA cycle for citrate biosynthesis [48]. The differential regulation of the proteins related to L-aspartate, fumarate (ADSS and ASL) (Table S2), and oxaloacetate biosynthesis indicate that Cd stress induces changes in oxaloacetate biosynthesis. Moreover, the downregulation of ATP-citrate synthase indicate that the conversion of oxaloacetate to citrate is probably reduced when *P. cyrtonema* is grown in Cd stress [49]. Since citrate application can alleviate Cd stress e.g., in rice [50], therefore, the DEPs presented above are ideal candidates for characterization in *P. cyrtonema* under Cd stress and to evaluate the changes in ATP generation and diversion of routes for citrate.

Sphingolipids act as signaling molecules in biotic as well as abiotic stresses. Plants maintain a strict regulation of the free long chain base (a building block of sphingolipids), which control the fate of cell i.e., apoptosis or survival via proliferation [51]. The downregulation of glycosphingolipid biosynthesis related proteins and the upregulation of PPP2RA1 and PLDα1 (Table S2) indicate that Cd stress could initiate sphingolipid signaling to affect apoptosis. However, limited knowledge is available on the role of sphingolipid signaling in heavy metal stress in plants [52] and these observations need further exploration. Nevertheless, studies involving PLDα1 have indicated its role in metabolism and RNA binding, protein import in chloroplast, accumulation of photosynthetic proteins, and chloroplast size and biogenesis [53]. Contrary to these, the downregulation of proteins enriched in flavonoid, alkaloid, and terpenoid biosynthesis indicate that Cd stress negatively affected the secondary metabolite biosynthesis. These results suggest that increased biosynthesis/application of compounds in these classes can possibly be utilized for Cd stress tolerance. Experiments in Arabidopsis have shown that application of flavonoids under Cd stress helped plants improve root length and seedling weight [54]. Apart from these, the changes in proteins associated with alpha-linoleic acid metabolism and fatty acid degradation indicate their essential roles in *P. cyrtonema* rhizome responses to Cd stress. Alpha-linoleic acid is a key factor in plant stress tolerance, as its content changes differently in plants with different sensitivities when challenged with abiotic stresses [55].

## Conclusion

**Fig. 6:**
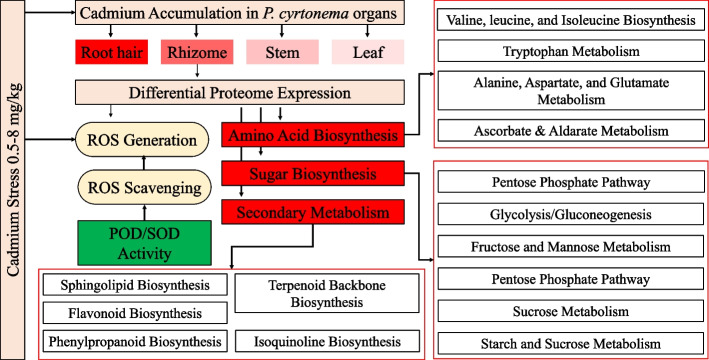
Summary of the effect of cadmium stress on *P. cyrtonema* tissues. The red color intensity in *P. cyrtonema* organs shows the level of cadmium. The red color on different pathways indicates that cadmium stress induces reduction of the expression of proteins related to these pathways. Green color indicates that the activity of the peroxidase (POD) and superoxidase dismutase (SOD) increased in *P. cyrtonema* rhizomes. Red outlines in different pathways indicate that cadmium stress significantly affected those pathways

Increase in soil Cd content from 0.5 to 4 mg/kg can increasingly accumulate in root hair, rhizome, stem, and leaf of *P. cyrtonema* plants. Whereas, a Cd concentration of 8 mg/kg is potentially damaging to the rhizomes. Different Cd concentrations affect the ROS generation and scavenging differently in rhizomes. The soil Cd content of 0.5 to 4 mg/kg affects the *P. cyrtonema* proteome in such a way the Cd concentration in growing medium is positively correlated with the number of DEPs i.e., the sever is the Cd stress, the higher is the number of DEPs. Cadmium stress changes the proteome profile of the *P. cyrtonema* rhizomes such that by increasing the concentration, the number of proteins (downregulated) involved, in sugar biosynthesis and related pathways, citrate cycle, alanine, aspartate and glutamate metabolism, valine, leucine and isoleucine biosynthesis, and secondary metabolism biosynthesis related pathways, increased. Taken together, Cd accumulation in the edible part (rhizome) of *P. cyrtonema* increases an increase in soil Cd concentration, which affects the primary metabolism, sugar biosynthesis, secondary metabolism, and defense related pathways (Fig. 6).

## Material and methods

### Plant material, growth conditions, and stress treatments

*P. cyrtonema* seeds were obtained from the Hunan Institute of Agricultural Environment and Ecology Genebank under the voucher number: HHGY120XXA. No prior permissions are required to work on this species. The seeds were identified by Prof Zi Li Yi (see author list). All relevant institutional, national, and international guidelines and legislations were followed while conducting this experiment. The experiment was setup in a triplicate completely randomized design. Plants were grown from seeds in pots filled with sandy loam soil with pH 6.0 ± 0.12, available Cd content of 0.04 mg/kg, and total Cd content was 0.14 mg/kg. Before filling the pots, the soil was dried and sieved, and 8 kg soil was filled into each plastic pot whose diameter and height are 12.5 cm and 17.5 cm, respectively. The plants were grown for three years. Standard agronomic practices were followed for allowing the plants to grow health.

Five treatments (including control) were set according to the total Cd concentration in the soil and each treatment consisted of 30 pots. As the national standard for soil Cd is 0.3 mg/kg, while farmland soils of Henan province contain 0.73 mg/kg Cd, therefore, we considered their average i.e., 0.5 mg/kg as a control (CK, hereafter D1). An increasing Cd concentration (gradient) was used as treatments D2 (1 mg/kg), D3 (2 mg/kg), D4 (4 mg/kg), and D5 (8 mg/kg). The soil Cd concentration was configured with CdCl_2_ according to mass ratio. After determining the Cd concentrations, the experimental pots were passivated for 90 days followed transplanting the three-years-old *P. cyrtonema* plants. The experimental plants were kept in a greenhouse for 90 days at the Hunan Institute of Agricultural Environment and Ecology, Mapoling, Furong District, Changsha City, China. The average temperature and humidity of the greenhouse were 25 ± 5 ℃ and 20%, respectively.

Three years old *P. cyrtonema* plants stressed with different levels of Cd were selected for further analyses. The plants were harvested, rhizomes (5–7 cm long with average weight of 25 g) separated, cleaned, and washed with distilled water thrice. The length of the rhizomes at the time of harvesting was 5–7 cm. Triplicate rhizome samples (25 g) were taken for each treatment. The harvested rhizomes were immediately frozen in liquid nitrogen and stored at -80 ℃ until further use for physiological indicators and proteome analyses. Additionally, root hairs, stems, and leaves were also harvested separately, washed, and used to determine Cd content. The Cd content in the *P. cyrtonema* organs was determined as reported in [56]. Since the rhizome (RP) is the edible part, therefore, it was used for further analyses i.e., the determination of activities of ROS scavenging enzymes and proteome analysis.

### Determination of enzyme activities

Triplicate rhizome samples were used for enzyme activity determination. All the procedures for activity determination were performed at -4 ℃. The rhizomes were homogenized with 2 mL 0.2 M phosphate buffer (pH 7.8), followed by centrifugation at 12,000 × g for 20 min, and the supernatant was taken for further assays.

The superoxide dismutase (SOD) activity was measured as reported earlier Zou et al. [57]. The absorbance was determined at 560 nm using a spectrophotometer (Model UV-1240, Shimadzu, Tokyo, Japan). One unit was defined as the amount of enzyme to inhibit 50% of the photochemical reduction of NBT.

Peroxidase (POD) activity was determined as follow: the mixture (3 mL) contained 0.1 mL supernatant, 0.13 M phosphate buffer (pH7.8), 13 mM hydrogen peroxide (H_2_O_2_), and 6.7 mM guaiacol, and one unit was defined as the amount of supernatant to increase the absorbance at 470 nm by 0.01 unit per minute.

Catalase (CAT) activity was determined as follow: the mixture (3 mL) included 0.1 mL supernatant, 0.2 M phosphate buffer (pH 7.8), and 13 mM H_2_O_2_, and the loss of absorbance at 240 nm was recorded at the first 30 s. One unit was defined as the amount of supernatant to decrease the absorbance at 240 nm by 0.01 units per minute.

Protein extraction, trypsin digestion, and tandem mass tags (TMT) labeling.

The rhizomes were taken out of -80 ℃ and ground in liquid nitrogen. 1 mL of extract was taken and added an equal volume of a saturated solution of phenol-Tris–HCl (7.8), followed by mixing at 4 ℃ for half an hour with periodic shaking. The mixture was centrifuged at 7100 xg for 10 min at 4 ℃ and supernatant was taken. Five volumes of pre-cooled 0.1 M ammonium acetate–methanol solution were added and kept overnight at -20 ℃. The next morning, the mixture was centrifuged at 12,000 xg for 10 min at 4 ℃, and the pallet was collected. Pre-chilled methanol (five volumes) was added, and the pallet was rinsed, mixed gently, and centrifuged at 12,000 xg for 10 min at 4 ℃. This process was repeated twice with acetone instead of methanol. The pallet was then dried at room temperature for five minutes, it was dissolved in lysis solution for three hours followed by centrifugation at 12,000 xg for 10 min at room temperature. The supernatant was taken, and the centrifugation step was repeated twice. The protein concentration was measured by the BCA protein determination method and stored at -80 ℃ [58].

For SDS–polyacrylamide gel electrophoresis, 10 µg protein was taken for each treatment and each replicate and separated by 12% SDS-PAGE, followed by staining with Coomassie brilliant blue and processed by following the method described by Candiano et al. [59].

For trypsin digestion, 50 µg protein from each sample (after determining protein concentrations) was taken and lysis buffer was used to dilute different groups of samples to the same concentration and volume. The solutions were then cooled on ice till room temperature was reached. Added iodoacetamide to make the final concentration of 9 mM, mixed well, and placed in the dark at room temperature for 15 min. To precipitate protein, 6 × acetone was added to the above solution and placed at -20 ℃ overnight followed by centrifugation at 8000 xg for 10 min at 4 ℃. Acetone was evaporated for 2–3 min and protein precipitates were collected. The pellet was reconstituted by adding 100 µL TEAB2, added 1 mg/mL trypsin Trypsin-TPCK at 1/50 of the sample mass, and digesting the sample at 37 ℃ overnight. This was followed by adjusting pH to 3 by adding phosphoric acid to terminate hydrolysis. Peptides were desalted by RP-C18 (Agilent) solid phase extraction column.

### LC–MS/MS analysis

Samples were detected by LC–MS/MS. Before MS injection, each sample was mixed according to the volume ratio of iRT i.e., sample to be tested = 1:10, as the internal standard. The iRT standard peptide was dissolved into a solution with a concentration of 10 × and stored it at 2–8 °C. For high pH liquid phase separation, the samples after enzymatic hydrolysis were mixed with equal amounts of peptide fragments, and the components were separated in the mobile phase of pH = 10 using the Agilent 1100 HPLC system. The separation conditions were: Column: Agilent Zorbax Extend – C18 narrow bore column, 2.1 × 150 mm, 5 μm, detection wavelengths: UV 210 nm and 280 nm, mobile phase A phase: ACN-H_2_O (2:98, v/v), mobile phase B phase: ACN-H_2_O (90:10, v/v) (both mobile phases were adjusted to pH 10 with ammonia water), flow rate: 250 μL/min, gradient elution conditions: 0–10 min, 2% B; 10–10.01 min, 2–5% B; 10.01–37 min, 5–20% B; 37–48 min, 20–40% B; 48–48.01 min, 40–90% B; 48.01–58 min, 90% B; 58–58.01 min, 90–2% B; 58.01–63 min, 2% B. For the component collection: starting from the 10th minute, the eluate was collected after every one minute into centrifuge tubes No. 1–10, and fractions were collected in the order of 1 → 10. A total of 10 components were collected, vacuum freeze-dried and drained, and the samples were cryopreserved until MS.

The MS scan parameters and liquid elution gradient are given in Table 2. The liquid elution gradient is shown in Table 3. The flow rate was 300nL/min, buffer A was 0.1% FA aqueous solution, and buffer B was 0.1% FA/80% ACN/20% water.

**Table 2:** DDA mass spectrometry conditions

**Items**	**Full MS**	**MS2**
**Resolution**	120000	30000
**AGC target**	3e6	2e5
**Maximum injection time**	100 ms	80 ms
**Scan range**	350–1650 m/z	200–2000 m/z
**Isolation window**	-	1.4 m/z
**Normalized Collision Energy**	-	27

**Table 3:** DDA chromatographic conditions

**Time (min)**	**Gradient**
**0**	4% B
**5**	4% B
**8**	9% B
**80**	28% B
**94**	44% B
**97**	90% B
**104**	90% B
**108**	5% B

The peptide fragments after enzymatic hydrolysis of each sample were collected separately on the computer. The scanning range was set to 350–1250 m/z, and the isolation window was 26 m/z. The DIA MS scan parameters are given in Table 4. The flow rate was 300nL/min, buffer A was 0.1% FA aqueous solution, and buffer B was 0.1% FA/80% ACN/20% water. The DIA chromatographic conditions are given in Table 5.

**Table 4:** DIA mass spectrometry conditions

**Items**	**Full MS**	**MS2**
**Resolution**	120 000	30 000
**AGC target**	3e6	1e6
**Maximum injection time**	100 ms	Auto
**Scan range**	350–1250 m/z	-
**Isolation window**	-	26 m/z
**Normalized Collision Energy**	-	28

**Table 5:** DIA chromatographic conditions

**Time (min)**	**Gradient**
**0**	4% B
**5**	4% B
**8**	9% B
**80**	28% B
**94**	44% B
**97**	90% B
**104**	90% B
**108**	5% B

### Data analyses

The original LC–MS/MS files were imported into Spectronaut Pulsar software (Biognosys AG, Wagistrasse, Switzerland) for database search and database construction. The main parameters were as follows. Missed cleavage (2), fixed modification (carbamidomethyl (C)), modification variable (Oxidation (M)), enzyme (Trypsin/P), protein FDR cut off (0.01), peptide FDR cut off (0.01), PSM FDR cut off (0.01), database (NCBI-asparagales.fasta). The DIA data analysis parameters were as follows. Precursor Qvalue cutoff (0.01), protein Qvalue cutoff (0.01), normalization strategy (local normalization), and quantity MS-level (MS2).

The trusted proteins were screened. The null values in the data matrix were replaced with half of the minimum value, data was log2 processed, normalized by the normalize/quantiles function in the package “preprocessCore”. The differentially expressed proteins (DATs) were screened if foldchange ≥ 1.5 and *p*-value < 0.05. The heatmaps were generated for the DATs and cluster analysis was done using unsupervised hierarchical clustering based on R. Pearson correlation coefficient and Principal component analysis (PCA) was calculated using protein expressions between the DATs. The proteins were functionally characterized according to UniProt [60], KEGG [61], GO [62], and KOG/COG [63] databases. Furthermore, GO/KEGG enrichment [64] analysis was performed on DATs.

## Supplementary Information

**Additional file 1: Supplementary Table 1.** Absorbance and concentration of the protein samples extracted from Cadmium treated Rhizoma Polygonati. **Supplementary Table 2.** List of differentially expressed proteins in cadmium treated Rhizoma Polygonati.

**Additional file 2: Figure S1.** GO enrichment of the differentially expressed proteins in different treatment comparisons of cadmium-treated Rhizoma Polygonati.

**Additional file 3: Figure S2.** KEGG pathway enrichment of the differentially expressed proteins in different treatment comparisons of cadmium-treated Rhizoma Polygonati.

## Data Availability

The datasets generated and/or analyzed during the current study are available in the iPROX repository: https://www.iprox.cn//page/project.html?id=IPX0005725000.
