# Supplementary material for: Comparative proteome profiles of Polygonatum cyrtonema Hua rhizomes (Rhizoma Ploygonati) in response to different levels of cadmium stress
Source: BMC Plant Biol. 2023 Mar 20;23:149. doi: 10.1186/s12870-023-04162-6 (PMC10026435; doi:10.1186/s12870-023-04162-6)
Supplement: Supplementary file 3 — Additional file 3: Figure S2. KEGG pathway enrichment of the differentially expressed proteins in different treatment comparisons of cadmium-treated Rhizoma Polygonati. [file 12870_2023_4162_MOESM3_ESM.pptx]

## Slide 1
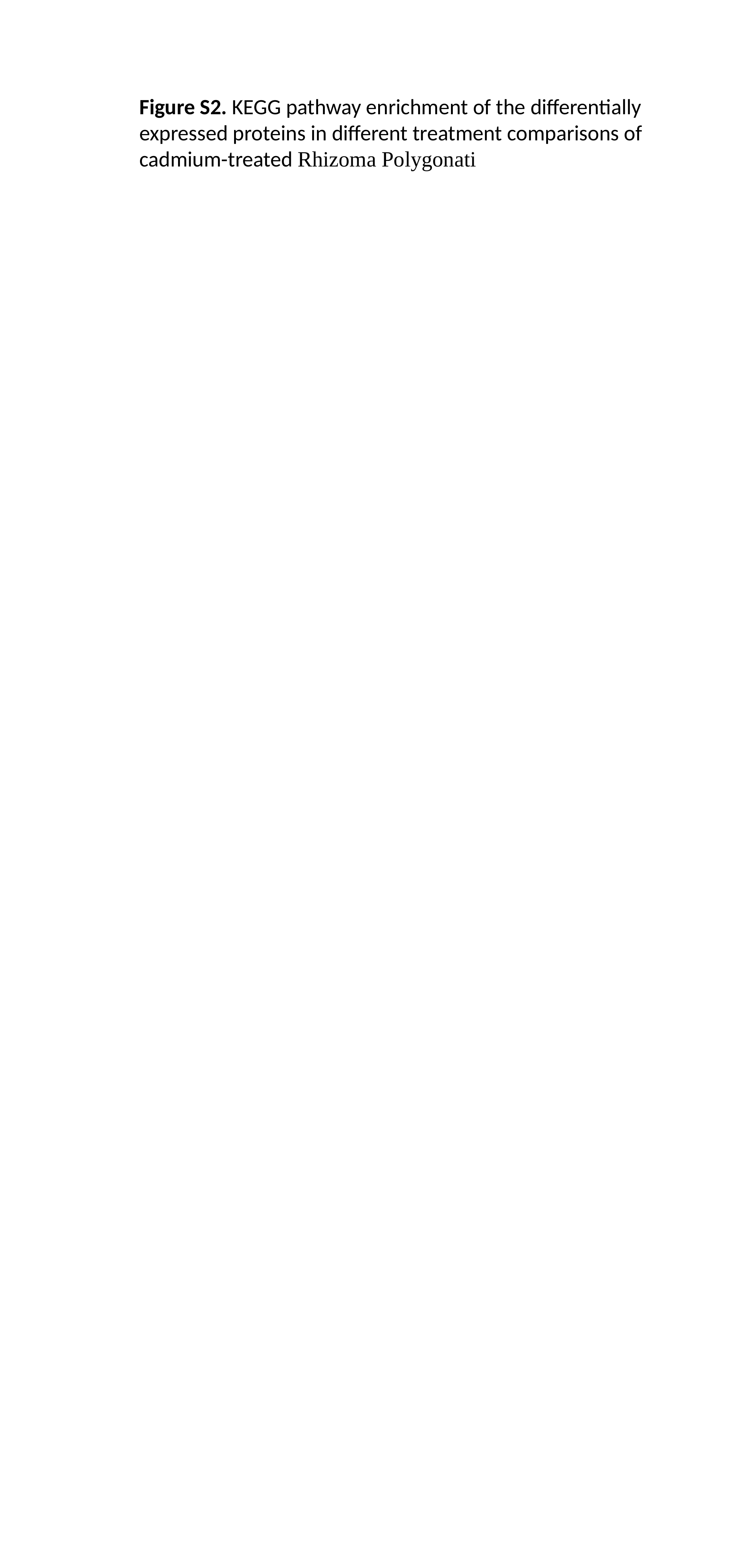

Figure S2. KEGG pathway enrichment of the differentially expressed proteins in different treatment comparisons of cadmium-treated Rhizoma Polygonati

## Slide 2
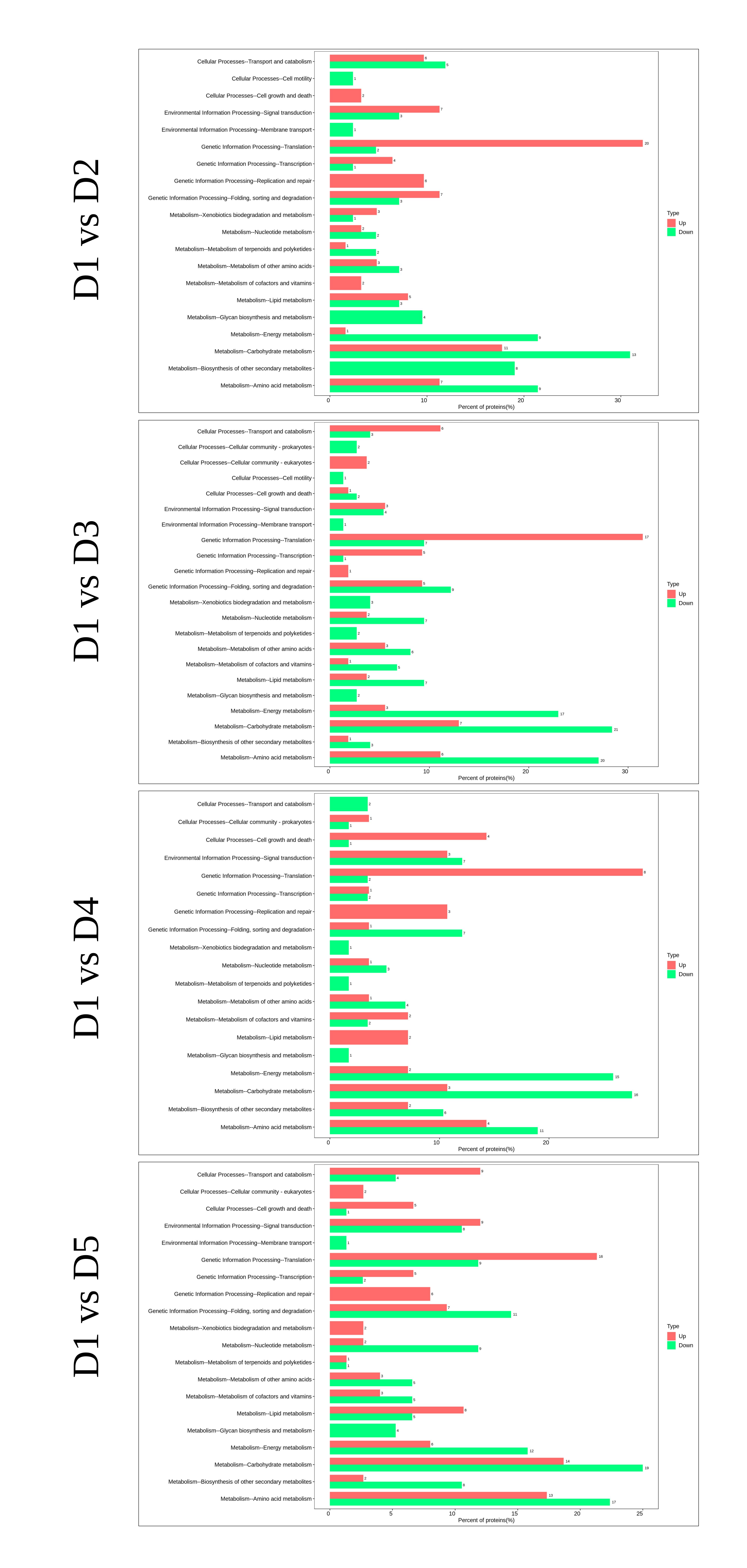

D1 vs D2
D1 vs D3
D1 vs D4
D1 vs D5
